# The Controversial Role of Glucocorticoids in Atheroembolic Renal Disease: A Narrative Review

**DOI:** 10.3390/jcm13216441

**Published:** 2024-10-27

**Authors:** Maria Chiara Pacchiarini, Francesca Di Mario, Paolo Greco, Enrico Fiaccadori, Giovanni Maria Rossi

**Affiliations:** 1 Department of Medicine and Surgery, University of Parma, 43126 Parma, Italy; 2 Nephrology Unit, University Hospital of Parma, 43126 Parma, Italy; 3 Laboratorio di Immunopatologia Renale “Luigi Migone”, University of Parma, 43126 Parma, Italy

**Keywords:** cholesterol crystal embolization (CCE), atheroembolic renal disease (ARD), acute kidney injury (AKI), crystallopathies, necroinflammation, glucocorticoids

## Abstract

Cholesterol crystal embolism (CCE) is an underrecognized multisystemic disease caused by the displacement of cholesterol crystals from atheromatous aortic plaques to distal vascular beds, leading to ischemic injury of target organs, particularly the kidneys, i.e., atheroembolic renal disease (ARD). According to recent research, cellular necrosis, induced by crystal-induced cytotoxicity, enhances the autoinflammatory cascade of the NLPR3 inflammasome, leading in turn to the so-called “necroinflammation”. The purported involvement of the latter in CCE offers a rationale for the therapeutic approach with anti-inflammatory drugs such as glucocorticoids, the use of which has long been a matter of debate in CCE. Diagnostic delay and no consistent evidence regarding efficacious treatment, leading to inconsistency in clinical practice, may worsen the already poor prognosis of ARD. The possible role of glucocorticoids in the treatment of ARD is thereby herein explored in a narrative fashion, analyzing the limited data from case reports and clinical trials.

## 1. Introduction

Cholesterol crystal embolism (CCE) is a life-threatening disease mainly due to the release of cholesterol crystals from ulcerated aortic plaques. Microemboli lodge in small-sized arteries, leading to ischemia and multiple organ dysfunction over time [1,2,3]. Like other conditions characterized by insidious and widely variable clinical presentation, CCE has been also labelled the “great masquerader” [4]. Atheroembolic renal disease (ARD), the sudden occlusion of small renal arteries by cholesterol crystals, is notably the most common and tricky-to-diagnose visceral involvement in CCE, with a reported incidence of 1–5% [5,6,7,8]. Indeed, it is often clinically silent and incidentally suspected late in the course of disease as worsening kidney function in patients with consistent clinical history. Acute kidney injury (AKI) is also a common scenario. Patients may also experience nonspecific symptoms such as flank pain, hypertension, and signs of systemic inflammation like eosinophilia. On the other hand, the skin is the most frequent extra-renal manifestation, recognizable as purple/blue toe syndrome or livedo reticularis. Skin biopsy is a valuable technique to reach a diagnosis of CCE [9,10,11]. Systemic involvement with transient ischemic attacks and stroke [12,13,14], retinal infarction (Hollenhorst plaques) [12,15,16,17], and gastrointestinal ischemia or bleeding [8,18,19] is less common.

CCE is a complication of severe atherosclerosis. Therefore, its main risk factors are the same: male sex, age > 60 years, dyslipidemia, smoking, diabetes, hyperuricemia, and hypertension [5,20]. The detachment of cholesterol crystals from ulcerated plaques may occur spontaneously due to mechanical stress, flow changes, inflammation, or intraplaque hemorrhage. Nevertheless, these precipitating factors are commonly a result of endovascular procedures (angiography or vascular surgery), anticoagulation, or polytrauma [20,21,22,23,24,25,26,27,28,29,30,31]. Moreover, since chronic kidney disease (CKD) is associated with advanced atherosclerosis, and patients with CKD are more susceptible for AKI, in course of excessive anticoagulation, they are more prone to develop anticoagulated-related nephropathy (ARN) concurrently with CCE [29]. Autopsy studies have reported a higher incidence of ARD in patients undergoing endovascular procedures/vascular surgery, on vitamin K antagonists (VKAs), and with risk factors for atherosclerosis (12–77%) than in the general population (1–5%) [5,6,7,8,11,32,33,34,35]. The estimated low incidence in clinical studies (1.4–0.09%) might therefore suggest that only a minority of cases become clinically apparent [36,37].

In the past few years, the widespread use of angiographic techniques and oral anticoagulants has contributed to an increasing interest in the recognition and treatment of this condition, which is associated with permanent disabilities and high mortality [29,30,33,36,38,39]. 

**Figure 1:**
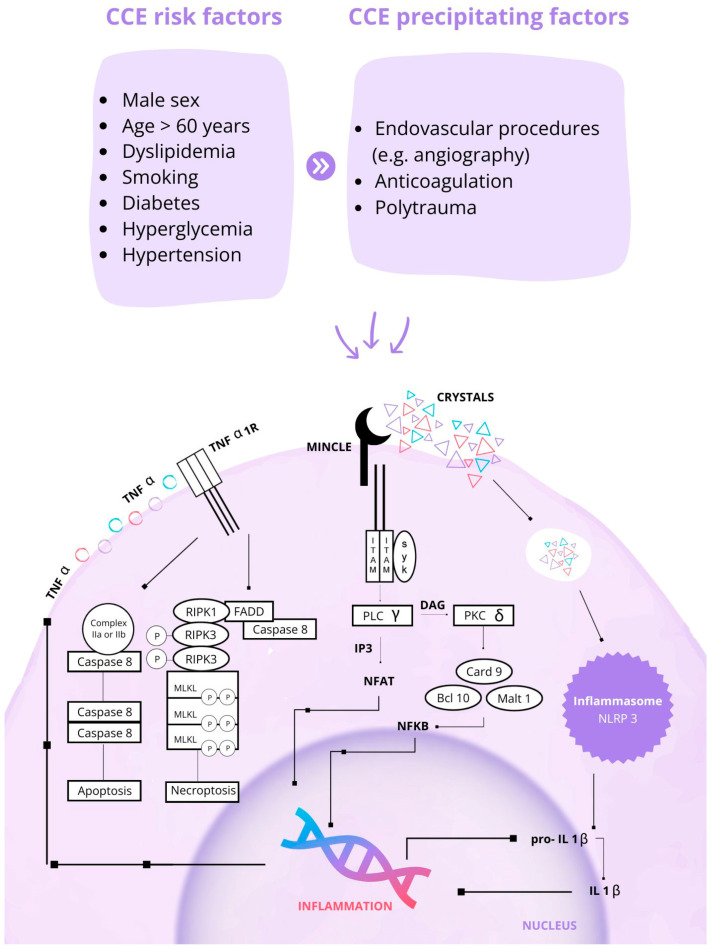
CCE risk factors and patterns of injury [27,39,40,41,44].

Recent advances in the understanding of its pathophysiology have led to the inclusion of CCE among “crystallopathies”. Indeed, the pathogenic mechanism underlying organ injury is only partially explained by vascular obstruction and ensuing organ ischemia, while the key role of an auto-inflammatory process has emerged [40]. The latter is mediated by TNF, NF-ĸB, and the inflammasome pathway (IL1β via NLRP3), leading to leukocyte infiltration with granulomatous reaction and interstitial fibrosis, referred to as “necroinflammation” [20,38]. Indeed, cholesterol crystals induce activation of interleukin (IL)-1β in mononuclear phagocytes via the nucleotide-binding and oligomerization domain (NOD)-like receptor protein 3 (NLRP3) inflammasome, which translates the danger signals into the enzymatic cleavage of pro-interleukin-1β (pro-IL-β) towards its mature form, causing cell necrosis. Moreover, cholesterol emboli induce the production of pro-IL-β via the activation of tumor necrosis factor receptor 1 (TNFR1) and the NF-ĸB pathway in macrophages and dendritic cells. Moreover, the atheroemboli directly adhere to the human macrophage-inducible C-type lectin (hMincle), releasing pro-inflammatory cytokines such as macrophage inflammatory protein 2 (MIP-2) and TNF [39,40,41]. The activation of several inflammatory pathways leads to the activation of different cellular death patterns called apoptosis and necroptosis, which are mediated by TNFR1 and RIPK1 (Figure 1).

Accordingly, anti-inflammatory drugs such as glucocorticoids might play a role in the treatment of ARD [42]. Recent advances of molecular research in the field of CEE-induced inflammation and microvascular thrombosis are centered on the activation of the complement cascade as a possible therapeutic target for CCE [43].

Although different studies have supported inflammation as a key mechanism in ARD, disagreements and concerns persist regarding the choice of treatment. Especially, the role of glucocorticoids in improving renal outcomes has not been confirmed yet despite wide use in clinical practice. The purpose of this narrative review is to evaluate the available evidence regarding the use of glucocorticoids in ARD, relying on the paucity of reported clinical experiences. 

## 2. Methods

We searched PubMed (https://pubmed.ncbi.nlm.nih.gov/, accessed on 30 September 2024) and Springer Link library (https://link.springer.com/, accessed on 30 September 2024) using the key words “atheroembolism”, “cholesterol embolism”, “cholesterol crystal embolisation”, “cholesterol crystal embolization”, and “atheroembolic renal disease”, individually and combined with “crystallopathies”, “necroinflammation”, “corticosteroids”, “glucocorticoids”, “acute kidney injury”, and “dialysis”, with a filter for studies published in the English language between 1999 and 2024, to identify relevant clinical studies, trials, and case reports suitable for our narrative review.

The inclusion criteria were the presence of (i) a clinical diagnosis with or without histological evidence (from kidney or skin biopsies) of ARD; (ii) AKI upon admission, according to AKI KDIGO guidelines criteria [45]; and (iii) a follow-up of at least two weeks after treatment with glucocorticoids with or without statins, with detailed renal outcomes. When histological diagnosis was not available, one or more of the following criteria had to be present: a recent history of high-risk endovascular procedures, pathognomonic ischemic manifestations of extremities such as blue toe syndrome or livedo reticularis, and Hollenhorst plaques on fundoscopy.

The exclusion criteria were the presence of other treatment for ARD, such as LDL-apheresis sessions or cyclophosphamide [46,47], and the lack of detailed follow-up, i.e., the lack of reporting of a key clinical outcome such as recovery of kidney function or lack thereof [48].

The main endpoint was full recovery of renal function after two weeks of treatment, in terms of a decrease in serum creatinine or an increase in estimated glomerular filtration rate (eGFR, mL/min/1.73 m^2^) back to baseline values [13,49]. Partial renal function recovery was defined by an increase in eGFR of at least 25% at two-to-four weeks after a diagnosis of ARD was reached. Secondary outcomes included (i) the resolution of systemic inflammation, assessed by decreasing C-reactive protein levels and eosinophil count, and (ii) the relapse of renal dysfunction after glucocorticoid withdrawal. Clinical relapse was defined as worsening renal function of any degree and increased eosinophil count and/or C-reactive protein. 

## 3. Results

We identified 18 eligible case reports [15,16,27,44,50,51,52,53,54,55,56,57,58,59,60] (Table 1) and a case series of 50 patients (32 treated with glucocorticoids) [61] (Table 2). A retrospective study on 345 patients (154 treated with glucocorticoids) did not meet our inclusion criteria due to the lack of follow-up data after treatment [62]. Fifty patients were treated with glucocorticoids and eleven with statin only, and these were therefore excluded. Thirty-one patients (31/50, 62%) were treated with both glucocorticoids and statins. We observed a low prevalence of spontaneous ARD (3/50, 6%) [48,61]. Many cases received a diagnosis of iatrogenic ARD (47/50, 94%), e.g., after angiography or cardiovascular procedures (27/50, 54%) and during antithrombotic therapy with heparin or VKAs (17/50, 34%), and, in three recent reports, DOACs (3/50, 6%).

The median age was 69 (range 55–82). All patients carried traditional cardiovascular risk factors. Almost all patients had pre-existing CKD (48/50, 96%), with a median eGFR of 40 mL/min/1.73 m^2^ (range 14–92). Organ damage mainly included renal dysfunction. However, skin involvement was also present in 33/50 (66%), while only 3 patients were screened for retinal plaques on fundoscopy, which was positive in all cases. Mild-to moderate eosinophilia (eosinophil count > 500 cell/mm^3^) was described in 44/50 (89%), and an increase in C-reactive protein levels was observed in 6/18 (33%) patients. AKI was present in all case reports and required dialysis in 2/18 (11%) patients. 

The prespecified outcomes are reported in Table 3 (case reports) and Table 4 (case series). Full renal function recovery was observed only in one patient (1/18, 6%). Partial renal function recovery was observed in 30/31 (97%) patients treated with both glucocorticoids and statins and in 8/19 (42%) of those treated with glucocorticoids only. Neither group had clinical relapses within two weeks of follow-up. At three months follow-up, two patients (one of the combined and the other of the glucocorticoid-only treatment group) experienced a rapid deterioration of renal function requiring hemodialysis, in conjunction with distal feet cyanosis and multiple lacunar cerebral infarctions in the first case and with serious intestinal ischemia in the second patient, which appears to be consistent with CCE recurrence. 

The great majority of case reports (38/50, 78%) were treated with daily 0.3 mg/kg oral prednisone, according to the protocol of Belenfant [25], a treatment that apparently led to clinical resolution of lower-limb and gastrointestinal lesions in CCE. No data about renal function improvement and prognosis were available in this study. Otherwise, more recent studies using a revised “Belenfant regimen” with a low-intermediate dose of oral glucocorticoids, e.g., 15 mg prednisolone or 25–50 mg prednisone for four weeks, showed good renal and global prognosis after 10–15 months of follow-up [15,27,42,44,50,51,53,58,59].

Glucocorticoid treatment regimens included (Table 2) low-intermediate daily oral prednisone/prednisolone 15–25 mg (around 0.3–0.4 mg/kg considering a mean weight of 70 kg) in 39 patients (39/50, 78%) and started upon diagnosis, with gradual tapering until a maintenance dose of 2.5–5 daily within 2–4 weeks, with a median overall duration of 6–12 months [15,27,44,50,53,58,61]. In six patients (6/50, 12%) a high-intermediate dose oral regimen (30–50 mg daily) was chosen, with gradual tapering over months until a maintenance daily dose of 5 mg was reached [51,56,59,63]. Five patients (5/50, 10%) were treated with a high-dose regimen, with two or three pulses of 250 mg intravenous methylprednisolone or daily 1 mg/kg oral prednisolone, followed by tapering until a maintenance dose of 20 mg [16,52,54,55,57]. In two cases, glucocorticoid pulses were administered in non-responders to a low-dose oral steroids regimen, achieving partial response. Three patients experienced a clinical improvement with an early treatment with pulses or a high oral prednisolone dose. Only one case reported a relapse after a six-month maintenance treatment. 

In terms of secondary outcomes, a significant decrease in the eosinophil count and in CRP levels was observed, both showing normalization upon treatment completion (39/50, 78%). However, in 7/18 (39%), a new increase in inflammatory markers (i.e., eosinophils and/or CRP), worsening in renal function, or a clinical relapse after glucocorticoid discontinuation were observed, either after a two-week glucocorticoid regimen or a six-month glucocorticoid treatment. Curiously, in nearly all cases of glucocorticoid withdrawal with laboratory and/or clinical relapse, a statin was not administered in association (6/7, 85.7%). 

Nakajama [61] retrospectively compared renal outcomes (using our same endpoints) between a group of 32 patients undergoing glucocorticoid therapy and a group of 19 untreated patients (Table 3). In the glucocorticoid-treated group, the increase in eGFR and the decrease in sCR after four weeks of treatment were significantly different and improved in comparison with those at the time of diagnosis, whereas in the untreated group, renal function amelioration was not statistically significant. Percent change per year in eGFR, however, was not statistically different between groups. The same applies for secondary endpoints: Eosinophil count was significantly reduced after four weeks of glucocorticoid treatment, while it was not reduced in the group not treated with glucocorticoids, with, however, no difference in % changes per year in eosinophil counts between groups. Moreover, while a combined therapy with statin in 25/32 patients had a favorable impact on eGFR after four weeks of treatment, in the long term, the % change per year did not.

## 4. Discussion

CCE is an insidious disease that is often forgotten because of the wide variability in clinical presentation. Treatment choices are controversial due to the lack of evidence and still rely on local practice. 

Our review focused on the role of glucocorticoid treatment in ARD. There is encouraging but thin evidence supporting their efficacy, derived from a few case reports, obviously highlighting the need for confirmation in large, randomized trials. 

The rationale for anti-inflammatory drugs is supported by emerging evidence on pathophysiology. Although the pathogenetic mechanism of ARD is still poorly understood, recent studies focusing on atherosclerotic plaque destabilization have identified the activation of a specific inflammation pathway referred to as “pyroptosis” as the cornerstone of vascular damage [64,65,66]. Pyroptosis is a recently described form of programmed cell death accompanied by an intense inflammatory response [66]. The lead role in this autoinflammatory cascade is played by the inflammasome. Specifically, cholesterol crystals in atherosclerosis act as the “Toll-Like receptor” of the innate response to pathogens, thus activating the inflammasome pathway. The NLP3 inflammasome enhances the activation of caspase-1, leading to the production of IL-1β and IL-18, thereby driving cells towards pyroptosis [64]. Translating the emerging role of the “necroinflammation” cascade triggered by crystals into the pathogenesis of AKI in the course of crystallopathies, recent studies have identified the NLP3 inflammasome pathway as implicated in oxalate and uric acid nephropathy [49,67]. CCE pathogenesis is complex and multifactorial: On the one hand, systemic inflammation sustains the destabilization of atherosclerotic plaques, and on the other hand, cholesterol microemboli occlude branches of kidney arteries, leading to ischemic injury and local inflammation with AKI [29,30,49,67,68]. In this scenario, “necroinflammation” is both a cause and a consequence of AKI. A two-fold rationale, both for glucocorticoids and statins, becomes straightforward based on these considerations [68,69]. Curiously, recent animal research pointed out hyperglycemia and hyperuricemia as aggravating risk factors for kidney ischemic injury connected, respectively, to necroinflammation and diffuse vasoconstriction in CCE [30]. Consequently, this might support a careful use of CCS by monitoring and correcting metabolic abnormalities. 

We put an emphasis on the possible role of glucocorticoids in the short-term period (two weeks of treatment) in the reduction in inflammation and improvement in organ failure. Indeed, we observed, both in case reports [16,27,28,31,44,51,52,53,54,58] and in the retrospective study by Nakayama [61], a relationship between the decrease in systemic inflammation and a positive trend of renal function recovery. On the other hand, the recurrence after withdrawal of glucocorticoid therapy and non-significant percent changes per year in terms of renal function recovery between the glucocorticoid treatment group and the untreated one in the latter study show that efficacy in the short term does not necessarily associate with long-term preservation of renal function. 

The clinical study by Scolari et al. [62] was excluded from the analysis because it did not meet our prespecified criteria but deserves detailed comments since it is the largest study on ARD published to date. Glucocorticoids were initiated in 154/354 (43.5%) of patients and a statin in 115/354 (32.4%), both combined with pentoxifylline. Detailed follow-up data on patients treated with glucocorticoids are not available (in terms of eGFR variations upon treatment). However, renal outcomes were generally poor. In fact, 116/354 (32.7%) patients required dialysis therapy. Statin treatment was significantly associated with a better renal prognosis, while glucocorticoids were not associated with outcomes. The protective benefit of statins was previously reported in a longitudinal cohort study [12], which showed that patients on statin treatment had a significant lower risk of developing end-stage kidney disease. The lack of benefit from glucocorticoids reported in this study is, however, hardly definitive. The study was not focused on a specific evaluation of CCE treatment, and the results could be affected by population selection bias. Included patients had clinically overt CCE syndromes, i.e., those with more severe disease and therefore possibly less responsive to treatment. In line with previous findings from the same group, pre-existing chronic renal impairment was identified as an independent variable associated with increased probability of end-stage kidney disease [12]. Finally, the great part of the study population suffered from severe cardiovascular disease and was therefore more prone to benefit from statin initiation in terms of overall survival. 

Regarding timing and dosing of glucocorticoid treatment, there was wide variability. In a previous report, Nakayama [61] underlined that early initiation of glucocorticoid therapy in CCE might improve outcomes. This might be correlated with the precocious cessation of the inflammatory reaction surrounding affected renal vessels, thus preventing the transition towards fibrosis, but such findings should be assessed in a more rigorous, perspective trial. To date, trials aimed at identifying the most effective glucocorticoid regimen in ARD are lacking. 

## 5. Limitations

This narrative review has many limitations. First, a selection bias exists because of the inherently “poor” literature about CCE, mainly relying on case reports and case series (either retrospective or perspective observational studies) with widely different treatment protocols and heterogenous populations. As a result, the comparison between large clinical studies and the single-patient experience reported by a case report is not feasible. Finally, the lack of long-term follow-up data in most of these studies represents another major limitation. 

## 6. Conclusions

Based on our short narrative review, we can define some key points. The pathogenetic mechanism of “necroinflammation” underlying organ damage in CCE serves as a solid rationale for the design of trials on the use of glucocorticoids, further supported by the apparent benefit from sparse case reports and case series. Secondly, since elderly people with multiple cardiovascular risk factors are the most exposed population, one must consider CCE as a potentially recurring/relapsing disease: As long as modifiable risk factors are still present, disease can and will recur, as suggested by those cases in which withdrawal of glucocorticoids resulted in deterioration of renal function [16,52,54,56,61].

Future therapeutic approaches might include complement blockade and therefore acting on inflammation and microvascular damage, but the current evidence is still preliminary [43].

For these reasons, the impact of glucocorticoid therapy on renal and global outcomes in CCE deserves to be established with randomized clinical trials built on a specific glucocorticoid treatment protocol and also evaluating the optimal timing of glucocorticoid initiation and treatment duration (i.e., the necessity or not of “maintenance” treatment), which implies the necessity of long-term follow-up data.

## Figures and Tables

**Table 1:** Demographic characteristics in selected case reports.

	Age	Sex	sCR Baseline	eGFR Baseline	Triggering Factor	CCE Organ Involvement	Diagnosis
Mann, et al., 2001 [51]	79	M	1.1	64	Endovascular procedures	Kidney, skin	Clinical
Graziani, et al., 2001 [16]	70	M	2.8	22	Endovascular procedures	Kidney, skin, retina	Clinical
Fabbian, et al., 1999 [54]	82	M	3.7	14	Endovascular procedures	Kidney, skin	Clinical
Nakahama, et al., 2001 [53]	55	M	2.1	34	Endovascular procedures	Kidney	Clinical
Desai, et al., 2011 [55]	57	M	1.2	67	Endovascular procedures	Kidney, skin	Histological
Takahashi, et al., 2003 [52]	65	M	0.9	85	Endovascular procedures	Kidney	Histological
Oka, et al., 2018 [27]	80	M	0.9	80	DOAC	Kidney, skin	Histological
Pistolesi, et al., 2018 [15]	81	M	1.6	40	Endovascular procedures	Kidney, skin, retina	Histological
Matzumura, et al., 2006 [50]	68	M	1.7	41	Warfarin	Kidney, skin	Histological
Koga, et al., 2005 [63] (patient 1)	77	M	1.7	38	DOAC	Kidney	Histological
Koga, et al., 2005 [63] (patient 2)	59	M	1.0	82	Endovascular procedures	Kidney, skin	Histological
Stabellini, et al., 2000 [56]	61	M	0.9	92	Endovascular procedures	Kidney, skin	Histological
Piranavan, et al., 2019 [57]	62	F	1.5	37	Spontaneous	Kidney	Histological
Pacchiarini, et al., 2022 [44]	69	F	1.5	35	DOAC	Kidney, brain	Histological
Masuda, et al., 2013 [58] (1)	70	M	2.0	33	Endovascular procedures	Kidney, skin	Histological
Masuda, et al., 2013 [58] (2)	68	M	1.6	45	Endovascular procedure	Kidney, skin	Clinical
Faria, et al. 2011 [59]	70	M	2.0	33	Endovascular procedure	Kidney, skin, brain, retina	Histological
Cheng, et al., 2022 [60]	76	M	-	-	Spontaneous	Kidney, skin	Histological

Abbreviations: sCR, serum creatinine (mg/dL); eGFR, estimated glomerular filtration rate (CKD-EPI eGFR mL/min/1.73 m^2^); CCE: cholesterol crystal embolism; M, male; F, female; DOAC, direct oral anticoagulant therapy.

**Table 2:** Demographics characteristics in Nakayama et al. [26].

Studies	Pt	Statins	Age	eGFR	Triggering Factor		CCE Organ Involvement		Diagnosis
Nakayama, et al., 1987 [26]	51	36	73.8 ± 6.8	26.6 (19.1–39.3)	Post angiography	Anticoagulation	Kidney	Skin	Clinical 16 Histological 35
Steroids (+)	32	25	74.0 ± 7.5	25.3 (17.2–43.8)	15	16	7	31	
Steroids (−)	19	11	73.5 ± 5.5	27.4 (26.0–39.3)	12	11	6	16	

**Table 3:** Outcomes of selected case reports.

	Starting Treatment						2-Week Treatment Follow-Up						Long-Term Follow-Up
	sCR	eGFR	Eo% (a.v)	CRP	Steroids ^+^	Statins ^+^	sCR	eGFR	Eo% (a.v)	CRP	Steroid Regimen Therapy	Relapsing Episode	sCr
Mann, et al., 2001 [51]	3.9	14	6	-	Yes	-	2.3	26	-	-	50 mg o.p.	No	-
Graziani, et al., 2001 [16]	4.0	14	-	50	Yes	-	3.0	20	-	0	25 mg po + 1 mg/kg IV mp + 0.5 mg/kg o.p	Yes	Amelioration after 3rd cycle (6 months)
Fabbian, et al., 1999 [54]	6.6	8	18 (1341)	100	Yes	No	5.1	10	(95)	4.6	250 mg IV mp + 50 mg o.p. + 25 mg p.o (2° regimen)	Yes	-
Nakahama, et al., 2001 [53]	10.5	5	23 (2378)	41.8	Yes	No	3.9	16	-	-	15 mg o.p.	Yes	-
Desai, et al., 2001 [55]	7.5	7	-	-	Yes	Yes	1.6	47	-	-	1 mg/kg oral mp	No	-
Takahashi, et al., 2003 [52]	3.0 and 6.5	21 and 8	12.6 (1160)	5	Yes	-	3.4	18	0	0	20 mg o.p.+ 1 mg/kg IV mp	Yes	3.5 (12 months)
Oka, et al., 2018 [27]	2.3	26	12 (2528)	175	Yes	No	-	30	-	0	15 mg o.p.	No	-
Pistolesi, et al., 2018 [15]	4.5	11	12.4	-	Yes	Yes	2.0	30	2.4	-	25 mg o.p.	No	-
Matsumura, et al. 2006 [50]	5.5	10	9 (999)	-	Yes	Yes	2.0	33	(500)	-	15 mg o.p.	No	-
Koga, et al., 2005 [63] (patient 1)	3.7	15	15 (934)	-	Yes	No	2.0	31	-	-	30 mg o.p.	No	-
Koga, et al., 2005 [63] (patient 2)	1.8	40	7 (428)	5	Yes	No	1.0	82	-	-	30 mg o.p.	No	-
Stabellini, et al., 2000 [56]	6.8	8	11.8 (1486)	85	Yes	No	2.4	28	-	-	35 mg o.p.	Yes	2.4 (ongoing treatment, 12 months)
Piranavan, et al., 2019 [57]	5.2	8	-	-	Yes	No	2.5	20	-	-	1 mg/kg IV mp + 40 mg o.p.	No	-
Pacchiarini, et al. 2022 [44]	4.3	10	11 (664)	-	Yes	Yes	3.1	15	0	-	25 o.p.	No	3.5 (6 months)
Masuda, et al. 2013 [58] (1)	7.1	7	5136	56	Yes	-	3.5	17	260	-	20 mg o.p.	No	3.5
Masuda, et al., 2013 [58] (2)	3.8	22	805	-	Yes	-	2.88	16	213	-	20 mg o.p.	Yes	death
Faria, et al., 2019 [59]	4.3	13	10	2.5	Yes	Yes	9.0	HD	-	-	30 mg o.p.	No	death
Cheng, et al., 2022 [60]	2.7	22	3460	-	Yes	Yes	2.5	24	3000	-	30 mg o.p.	No	1.6 (12-month treatment)

Abbreviation: sCR, serum creatinine (mg/dL); eGFR, glomerular filtration rate (mL/min/1.73 m^2^); Eo, eosinophils (% or count cell/mm^3^); CRP, C-reactive protein (mg/L); HD, hemodialysis; IV, intravenous injection; mp, methylprednisolone; o.p., oral prednisolone/prednisone.

**Table 4:** Study outcomes in Nakayama et al. [61].

**Study Population**	**Diagnosis**				**4 Weeks of Treatment**		**Last Follow-Up**			
	**sCR**	**eGFR**	**Eo**	**CRP**	**sCR**	**eGFR**	**sCR**	**eGFR**	**Eo**	**CRP**
	2.9 (2.1–4.0)	17.0 (12.0–24.6)	677	8.7 (1.6–30.4)	2.3 (1.8–3.0)	21.2 (16.9–29.0)	2.5 (1.8–3.5)	19.3 (13.9–28.7)	230	3.9 (0.8–21.3)
Glucocorticoid-treated group	3.0 (2.2–4.3)	25.3 (17.2–43.8)	637	7.6 (0.9–20.0)	2.4 (−18%)	20.6 (+24%)	2.5 (−8%)	19.3 (+11%)	170 (−42%)	2.5 (−22%)
Untreated group	2.9 (2.0–4.0)	27.4 (26.0–39.3)	808	8.7 (2.4–74.1)	2.2 (−5%)	24.7 (+5%)	2.5 (−5%)	20.1 (+11%)	454 (−18%)	4.9 (−13%)

Abbreviations: Pt, patients; sCR, serum creatinine (mg/dL); eGFR, estimated glomerular filtration rate (mL/min/1.73 m^2^); Eo, eosinophils (count cell/mm3); CRP, C-reactive protein (mg/L).
